# Development of high-throughput systems for biodosimetry

**DOI:** 10.1093/rpd/ncad060

**Published:** 2023-09-18

**Authors:** Ruth C Wilkins, Lindsay A Beaton-Green

**Affiliations:** Consumer and Clinical Radiation Protection Bureau, Health Canada, Ottawa K1A 1C1, Canada; Consumer and Clinical Radiation Protection Bureau, Health Canada, Ottawa K1A 1C1, Canada

## Abstract

Biomarkers for ionising radiation exposure have great utility in scenarios where there has been a potential exposure and physical dosimetry is missing or in dispute, such as for occupational and accidental exposures. Biomarkers that respond as a function of dose are particularly useful as biodosemeters to determine the dose of radiation to which an individual has been exposed. These dose measurements can also be used in medical scenarios to track doses from medical exposures and even have the potential to identify an individual’s response to radiation exposure that could help tailor treatments. The measurement of biomarkers of exposure in medicine and for accidents, where a larger number of samples would be required, is limited by the throughput of analysis (i.e. the number of samples that could be processed and analysed), particularly for microscope-based methods, which tend to be labour-intensive. Rapid analysis in an emergency scenario, such as a large-scale accident, would provide dose estimates to medical practitioners, allowing timely administration of the appropriate medical countermeasures to help mitigate the effects of radiation exposure. In order to improve sample throughput for biomarker analysis, much effort has been devoted to automating the process from sample preparation through automated image analysis. This paper will focus mainly on biological endpoints traditionally analysed by microscopy, specifically dicentric chromosomes, micronuclei and gamma-H2AX. These endpoints provide examples where sample throughput has been improved through automated image acquisition, analysis of images acquired by microscopy, as well as methods that have been developed for analysis using imaging flow cytometry.

## Introduction

Biomarkers for ionising radiation exposure have great utility in determining if an exposure has occurred. Some biomarkers, which respond as a function of dose, can be used as biodosemeters to determine the dose of radiation to which an individual has been exposed when physical dosimetry is missing or in dispute. Currently, there are many biomarkers under evaluation^(1)^ for emergency response ranging from molecular biological endpoints^(2)^, DNA damage and repair^(3)^, chromosome damage^(4)^ and physical methods such as electron paramagnetic resonance or optically stimulated luminescence^(5, 6)^. Each of these techniques are at different levels of maturity and have applications in different scenarios^(7–10)^. The most traditional biomarkers are cytogenetic biomarkers such as the measurement of dicentric chromosomes (dicentric chromosome assay (DCA))^(11, 12)^ or micronuclei (MN) in binucleated cells (cytokinesis-block micronucleus (CBMN) assay) as a measure of dose^(13–16)^. These two assays are the most commonly used biodosimetry methods worldwide^(17)^. Additionally, the measurement of the phosphorylated histone variant H2A.X (gamma-H2AX assay), a marker of DNA double-strand breaks, is gaining popularity as a biomarker of exposure^(18–20)^. This assay is characterised by a signal which appears within minutes of exposure, peaks around 1-hr post-exposure and then returns to near background levels by 48 hrs. For this reason, the applicability of gamma-H2AX is limited as a biodosemeter to those situations where samples can be fixed within hours of exposure. Nonetheless, the gamma-H2AX assay may be useful as a biomarker of exposure, indicating that an exposure has occurred without being able to provide an accurate dose estimate. This would be extremely useful as a triage tool to rapidly identify those individuals who have not been exposed and do not need any further analysis^(21)^. This review will focus on these three assays, which have advantages and disadvantages as listed in Table 1.

**Table 1:** Advantages and disadvantages of a selection of traditional (microscope-based) biodosimetry assays.

Assay	Advantages	Disadvantages
DCA	Radiation-specific Dose-responsive 0–5 Gy dose range Internationally standardised Low background signal	48 hr culture period labour-intensive Requires expert knowledge to score
CBMN Assay	Ease of scoring Dose-responsive 0–5 Gy dose range Internationally standardised	72 hr culture period Variable background Age/sex/lifestyle effects
Gamma-H2AX	No culture period required Signal appears within minutes Radiation-specific	Rapidly changing signal over time Not accurate as a biodosemeter

Traditionally, these assays have been analysed by microscopy, a process that can be time-consuming and labour-intensive. For accurate biodosimetry, many cells need to be analysed (~1000 cells), especially for lower doses. This is feasible for individual dose estimates but can become an issue for large-scale events where hundreds or thousands of individuals may require biodosimetry analysis. In these mass casualty scenarios providing rapid dose estimates to the medical community in a timely manner is essential to inform medical interventions that could mitigate the effects of acute radiation syndrome. Furthermore, there could be many non- or low-exposed individuals near the event who would benefit from biodosimetry to help alleviate the psychological effects of potential radiation exposure. In these cases, traditional manual microscope-based analysis for biodosimetry is no longer feasible and methods for high-throughput analysis are required.

Many steps of the biodosimetry process are amenable to automation, starting from sample processing^(22–26)^, through to data acquisition and image analysis. There are also many technologies for the automated analysis of slides, including laser scanning cytometry ^(27)^. However, this manuscript will focus on a review of data acquisition and image analysis using metaphase finders, imaging flow cytometers and the image analysis that accompanies both image acquisition technologies.

## Slide-based analysis

**Figure 1:**
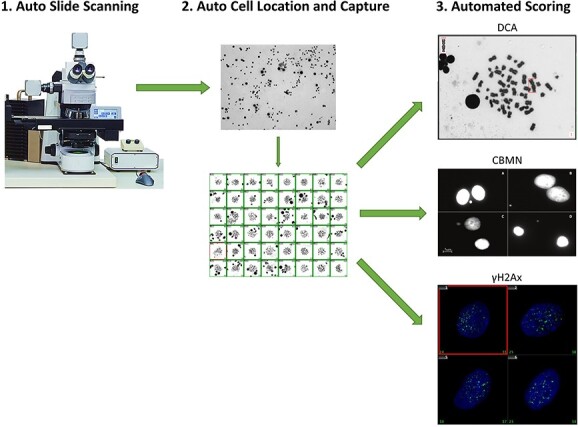
Schematic of process for automated cell location and image analysis of damage.

When blood samples are received at a biodosimetry laboratory for analysis, they must be processed according to the established methods corresponding to those used to generate calibration curves in that laboratory. This typically entails samples that are fixed and dropped onto slides for microscope-based analysis. Once slides have been made, one of the most time-consuming aspects of data analysis is locating the cells of interest on the slide. These can be metaphase spreads in the case of the DCA, binucleated cells in the case of the CBMN assay or lymphocytes for gamma-H2Ax. This process can be expedited with the use of automated slide scanning microscopes which use algorithms to scan a slide, identify the cells of choice according to pre-determined cell classifiers, and record their coordinates on the slide such that they can be easily relocated^(28, 29)^. Once a slide has been scanned for the location of each cell, this data is saved such that each identified cell of interest can be revisited in order to visualise or capture a high-resolution image for either manual or automated image analysis (Figure 1)^(30)^.

For the DCA, there have been many examples of the use of image analysis for the identification of dicentric chromosomes with a simple monochromatic Giemsa stain. One of the first demonstrations of this technique noted a high number of false positive dicentrics, especially in the low-dose region, making it difficult to generate a useful calibration curve^(28)^. This was improved by adding a simple and quick manual verification of the dicentrics, by a trained user, which resulted in a low-background rate of dicentrics allowing a calibration curve to be created^(31)^. In this study, the calibration curve generated by automated dicentric analysis with manual verification (semi-automated) was compared to manual scoring, demonstrating that the manual calibration curve was much steeper than the semi-automated analysis. This indicates that the human eye is better at identifying the dicentric chromosomes than the machine. Similar analysis has been repeated independently by several laboratories^(15, 31–33)^.

This type of analysis has also been applied to the CBMN assay. Varga *et al.* first demonstrated a linear correlation between the automated count of micronuclei in binucleated cells and the manual count^(34)^. The slope of this correlation was less than one, indicating that, as with the DCA, the manual count detected more damage than the automated count. Also, similar to the DCA, there was a higher background rate of MN with the automated count, indicating an issue with false positive MN detection. Since this first analysis, many laboratories have developed automated or semi-automated calibration curves with comparisons to manual curves^(35, 36)^. The manual verification of automatically scored MN was found to reduce the false negative rate and improve the identification of missed MN. This semi-automated analysis increased the sensitivity of the assay and increased the slope of the calibration curve, however, not as drastically as with DCA^(37)^.

The microscope-based method for the gamma-H2AX assay is also amenable to automated image analysis^(38, 39)^. In this case, the number of foci can be detected on slides after fluorescent labelling with anti-gamma-H2AX antibodies^(20, 40)^. The foci appear within minutes of exposure, peaking between 30 and 60 min and returning to near background levels within hours^(20)^. This technique is also applicable to other radiation-responsive foci such as 53BP1 or RAD51^(41)^.

Although automated slide scanning microscopes often have built-in image analysis algorithms for detecting damage in cells, there are also stand-alone systems that perform image analysis on images captured by these microscopes. These systems take image galleries acquired from automated slide scanning microscopes, select the optimal images, rank them according to quality and then analyse the best cells or metaphase spreads for damage^(42)^. The image galleries can range from uncurated (including all images without manual assessment) to highly curated, where a user has presorted the images to only include high-quality cells or metaphases. In each image, the software can then identify and enumerate the damage. In this manner, laboratories can establish calibration curves using these systems, and then similarly use them to generate dose estimates on an unknown sample based on the previously generated calibration curves. These types of systems have the potential for extremely high sample throughput and have been shown to be many times faster than semi-automated analysis using metaphase finding software, especially with the use of cloud-based parallel computing^(43, 44)^.

## Flow cytometry

Flow cytometry is a useful tool for high-throughput assays given its ability to analyse thousands of cells per minute directly from cell suspension without the time-consuming need to make slides. Not all biodosimetry methods, however, are well suited for traditional flow cytometry. For example, while sample preparation protocols for fluorescently labelling both chromosomes and centromeres released from metaphase cells have been developed, conventional flow cytometers struggle to detect the small differences in centromere fluorescence between a monocentric and dicentric chromosome as required for the DCA. Furthermore, aggregated chromosomes can easily be misidentified as dicentrics^(45, 46)^.

There has been somewhat more success with the development of a micronucleus assay using traditional flow cytometry^(47–49)^, which depends on using fluorescence intensity alone to differentiate between debris and micronuclei. As with automated microscopy, these methods have typically resulted in high false-positive rates. Additionally, the necessity of lysing the cells to release the nuclei and micronuclei into suspension eliminates the ability to use binucleated cells as a control for cell proliferation.

The gamma-H2AX assay, however, has been very successfully adapted to traditional flow cytometry. As the number of gamma-H2AX foci increases with dose, so does the overall intensity of the fluorescently labelled gamma-H2AX^(19, 50, 51)^. In fact, the intensity of the gamma-H2AX signal has been shown to increase monotonically up to 100 Gy^(52)^. This method has been applied to biodosimetry^(21, 40)^, though the rapidly changing kinetics of the assay remains a limiting factor, and the assay is best used as a biomarker of exposure.

## Imaging flow cytometry

Imaging flow cytometry (IFC) is a technique that combines the quantitative imaging analysis capabilities of microscopy with increased sample throughput and statistical power and fluorescence sensitivity of traditional flow cytometry^(53)^. With a potential rate of flow of 5000 events per second, the imaging flow cytometer has powerful implications to increase the sample throughput of traditional radiation biodosimetry techniques.

The adaptation of biodosimetry techniques to IFC was thoroughly reviewed in 2017, describing the challenges and successes of the DCA, CBMN and gamma-H2AX^(54)^. Some success has been seen with the DCA. However, there is still room for improvement in the processing of the samples to generate a solution of chromosomes that maintain their morphology through manipulation and do not become aggregated^(55, 56)^.

On the other hand, there has been considerably more progress and success in developing the CBMN assay for IFC. The sample processing has been well established^(57, 58)^ and there is continuing research on improving sample throughput with smaller volumes and shorter culture times^(59, 60)^. Furthermore, work is continuing to improve the dose estimate and extending the range of applicability through multiparametric analysis^(61)^. Finally, image analysis algorithms continue to improve through the establishment of robust templates and the addition of machine learning and artificial intelligence^(59, 62, 63)^.

The first instance of the gamma-H2AX being adapted to IFC was by Bourton *et al.* in 2012 who demonstrated a linear response for intensity of the gamma-H2AX as a function of dose upto 8 Gy with a similar kinetic response as demonstrated in earlier gamma-H2AX intensity studies^(64)^. With IFC, however, gamma-H2AX foci could also be quantified similarly to microscopy-based analysis. Similar to other automated analyses, the number of foci detected by IFC was, however, lower than that detected by microscopy. More recently, gamma-H2AX-labelling has been combined with 53PB1 detection. By analysing the co-localisation of these two DNA damage markers, the sensitivity of the assay can be improved by reducing the rate of false-positive foci^(65)^.

## Performance during inter-laboratory comparisons

While it is essential for a successful biodosimetry method to have a reproducible and robust dose-response calibration curve, the true test of a validated assay is not the steepness of the curve, but the ability of a measured biomarker to be translated to the dose of radiation received by the individual or sample. Many of the assays described above have been validated through inter-laboratory comparisons (ILC).

Automated and semi-automated microscope-based assays have been well validated through ILCs, particularly in those led by Running the European Network of Biological and retrospective Physical dosimetry (RENEB). A summary of some recent ILCs is compiled in Table 2. Semi-automated DCA analysis was conducted in several of the ILCs and has been shown to produce dose estimates comparable to manual microscope-based methods, successfully estimating the physical dose within a predetermined range of acceptability. Both fully and semi-automated CBMN analyses were conducted alongside manual analysis with great success. Dose estimates based on all analysis methods were in good agreement with the true dose. However, manual and semi-automated performed slightly better than fully automated analysis. As for gamma-H2AX analysis, manual versus automated analyses were compared in two RENEB ILCs. In both cases, the manual analysis performed better than the automated analysis. With gamma-H2AX, however, as it is less accurate in providing dose estimates due to the quickly changing signal, even manual analysis was only able to provide accurate rapid categorisation into low, medium and high dose ranges.

**Table 2:** Summary of selected inter-laboratory comparisons testing automated biodosimetry assays.

ILC	Assay	Image analysis method	# labs	# doses	Dose range	Results	Reference
NATO	DCA CBMN	Manual Semi-automated Manual Semi-automated Automated	6 3 3 2 3	10	0–6.4 Gy	MAD = 0.16–0.5 Gy MAD = 0.2–0.5 Gy excluding 6.4 Gy dose	(71) (34)
Multibiodose EU FP7	DCA CBMN Gamma-H2AX	Semi-automated Semi-automated Automated Manual Automated	6 5 5	4 8 4	0–4 Gy, acute, protracted and partial body 0–2.75 Gy, whole and partial body 0–4 Gy acute, protracted and partial body	Most doses within 0.5 Gy of given dose 100% accurate triage categorisation Automated had larger uncertainties	(66) (37) (68)
1st RENEB	Gamma-H2AX	Manual Automated	8 3	5	0–4 Gy	Accurate triage categorisation with manual but not automated scoring	(72)
2nd RENEB	CBMN Gamma-H2AX	Manual, Semi-automated Automated Manual Automated	12 8 3	4 2	0–4.75 Gy, whole and partial body 0.5 and 2.5 Gy	Good agreement with true dose. Semi-automated better than automated Most doses correctly categorised, manual performed better than automated	(67) (73)
4th RENEB	DCA	Manual Semi-automated	11 6	4	0–2.2 Gy	Little difference between manual and semi-automated	(74)

Some of the ILCs also evaluated the ability of the automated analyses to detect partial body (PB) exposures. In the Multibiodose ILC, the semi-automated scoring for DCA was evaluated for a simulated 50% PB exposure for three doses (2, 4 and 6 Gy). The detection of PB exposure was dependent on the dose, increasing from 32% at 2 Gy to 87% at 6 Gy. However, given that only 150 cells were analysed per sample, this would likely improve with higher cell numbers^(66)^. For CBMN, in the Multibiodose ILC, it was determined that semi-automated analysis was required over fully automated analysis to enable the detection of PB exposure^(37)^. However, in the 2nd RENEB ILC, all scoring methods detected the sample that was a simulated 50% PB irradiation^(67)^. For gamma-H2AX, attempts to identify PB exposures with automated microscope-based image analysis were not successful^(68)^. A more detailed list of ILCs using gamma-H2AX can be found in Raavi *et al.*^(21)^.

One study also examined whether accurate dose estimates could be achieved using semi-automated DCA after protracted exposures. It was determined that by applying the G-function^(69)^ to take into account the duration of exposure, all laboratories were able to distinguish between doses 1, 2 and 4 Gy with about half the dose estimations being within 0.5 Gy of the actual dose^(66)^. For the gamma-H2AX assay, it was determined that, while recent protracted exposures could be detected, it was difficult to quantify the dose received^(68)^.

## Conclusions

There has been great progress in increasing the sample throughput of biomarkers, notably biodosimetry, analysis for mass casualty situations. Although calibration curves for automated and semi-automated analyses tend to have a lower slope and a higher false-positive rate, when tested with blinded samples through ILCs, the dose estimates were comparable to those generated using manual analysis. Due to the shallower calibration curves, however, there could be loss of sensitivity along with an increase in the uncertainty of the dose estimates which could be mitigated by the ability to score many more cells. As long as the same method for generating both calibration curves and the dose estimates is used, the dose estimates should be accurate enough for rapid assessment for emergency response.

Through the development of biodosimetry networks, researchers have been able to collaborate on methods development projects followed by testing the newly developed assays in ILCs organised within and between these networks. There is still much potential for further improvement of these methods, including the addition of fluorescent labels to cytogenetic samples^(70)^ and the continued development of artificial intelligence and machine learning tools to assist in image analysis^(63)^. With the development of these tools, the sharing of technology and the co-operation that has developed within the international biodosimetry community, there is clear evidence of improved preparedness for mass casualty scenarios involving radiological and nuclear material.
